# Oligodendrocyte origin and development in the zebrafish visual system

**DOI:** 10.1002/cne.25440

**Published:** 2022-12-07

**Authors:** Adrián Santos‐Ledo, Cristina Pérez‐Montes, Laura DeOliveira‐Mello, Rosario Arévalo, Almudena Velasco

**Affiliations:** ^1^ Department of Cell Biology and Pathology, Instituto de NeurocienciasdeCastilla y León (INCyL) Universidad de Salamanca Salamanca Spain; ^2^ Instituto de Investigación Biomédica de Salamanca (IBSAL) Salamanca Spain

**Keywords:** development, myelination, oligodendrocytes, Sox, visual system, zebrafish

## Abstract

Oligodendrocytes are the myelinating cells in the central nervous system. In birds and mammals, the oligodendrocyte progenitor cells (OPCs) originate in the preoptic area (POA) of the hypothalamus. However, it remains unclear in other vertebrates such as fish. Thus, we have studied the early progression of OPCs during zebrafish visual morphogenesis from 2 days post fertilization (dpf) until 11 dpf using the *olig2:EGFP* transgenic line; and we have analyzed the differential expression of transcription factors involved in oligodendrocyte differentiation: Sox2 (using immunohistochemistry) and Sox10 (using the transgenic line *sox10:tagRFP*). The first OPCs (*olig2:EGFP*/Sox2) were found at 2 dpf in the POA. From 3 dpf onwards, these *olig2:EGFP*/Sox2 cells migrate to the optic chiasm, where they invade the optic nerve (ON), extending toward the retina. At 5 dpf, *olig2:EGFP*/Sox2 cells in the ON also colocalize with *sox10:tagRFP*. When *olig2:EGFP* cells differentiate and present more projections, they become positive only for *sox10:tagRFP*. *olig2:EGFP/sox10: tagRFP* cells ensheath the ON by 5 dpf when they also become positive for a myelin marker, based on the *mbpa:tagRFPt* transgenic line. We also found *olig2:EGFP* cells in other regions of the visual system. In the central retina at 2 dpf, they are positive for Sox2 but later become restricted to the proliferative germinal zone without this marker. In the ventricular areas of the optic tectum, *olig2:EGFP* cells present Sox2 but arborized ones *sox10:tagRFP* instead. Our data matches with other models, where OPCs are specified in the POA and migrate to the ON through the optic chiasm.

## Graphical Abstract

The first oligodendrocyte progenitor cells (OPCs, *olig2:EGFP*/Sox2, yellow cells) are detected in the preoptical area of the hypothalamus at 2 dpf. One day later, 3 dpf, these cells invade the optic chiasm and colonize the postchiasmatic region first and the prechiasmatic later (yellow cells). As they differentiate to oligodendrocytes, OPCs present more protrussions that wrap the optic nerve (ON) and become positive also for *sox10:tagRFP* (cyan cells). These triple‐labeled cells are very abundant in the ON at 5 dpf. At 7 dpf, differentiated oligodendrocytes (*olig2:EGFP*) with long protrusions that ensheath the ON are only positive for *sox10:tagRFP* and not for Sox2 (white cells). The three different populations can be observed surrounding the ON. At 11 dpf, the enseathing process continues. The first evidence of myelination in the ON is at 5 dpf, based on our transgenic line *mbpa:tagRFPt* (blue square patterned cells). We have also found that Olig2 and Sox2 might play a role in the differentiation of other retinal cells including the Müller glia and that the oligodendrocytes in the optic tectum differentiate earlier than those in the ON.

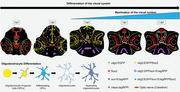

## 1 INTRODUCTION

Oligodendrocytes are the myelinating cells in the central nervous system (CNS) of vertebrates. Among other functions, they form the myelin sheath required for the fast saltatory conduction of nerve impulse (Baumann & Pham‐Dinh, 2001; Czopka, 2016; Hines, 2021). The visual system, as an intrinsic part of the CNS, is not an exception to myelination (Reichenbach et al., 1988), although there are some variations between species. For example, the ganglion cells axons that form the optic nerve (ON) are myelinated within the retina in fish (Fujita et al., 2000; Lillo et al., 2002; Münzel et al., 2012; Parrilla et al., 2016), reptiles (Santos et al., 2006), birds (Nakazawa et al., 1993), and some mammals like rabbits (Morcos & Chan‐Ling, 1997). However, in other mammals, including humans, the interior of the retina is not myelinated and its aberrant myelination is associated with age and causes several conditions (Berry‐Brincat & Shafquat, 2008; FitzGibbon & Nestorovski, 1997; Perry & Lund, 1990). Deficient myelination specifically in the visual system can also lead to several diseases known as Neuromyelitis Optica Spectrum Disorder (Berry‐Brincat & Shafquat, 2008). Thus, it is crucial to understand the first moments of myelination. Zebrafish is a well‐established model to study development and oligodendrocytes share many key transcriptional factors and specification routes between mammals and fish (Buckley et al., 2008; Lyons & Talbot, 2014; Mathews & Appel, 2016; Preston & Macklin, 2015).

Oligodendrocytes arise from the oligodendrocyte progenitor cells (OPCs), which are characterized by several transcription factors, including Olig2, Sox2, and Sox10 (Park et al., 2002; Takada & Appel, 2010; Takada et al., 2010). OPCs are highly mobile and proliferative cells, which migrate and search for their target axons along the nervous tracts until they ensheath them. In that moment, OPCs become myelinating oligodendrocytes and present, along with Olig2 and Sox10, myelin proteins such as Mbpa, Mpz, or Claudin Κ (Jung et al., 2010; Münzel et al., 2014; Nawaz et al., 2013). In vertebrates like chickens and mice, OPCs in the visual system are originated in the preoptic area (POA), from where they migrate to the optic chiasm (Bribián et al., 2006; Gao & Miller, 2006; Klionsky et al., 2021; Ono et al., 2017). However, the place where these OPCs originate in teleosts is not clear (Tian et al., 2016). Furthermore, the initiation of the myelination process during development is not fully understood. For example, although the first evidence of myelination (*mbpa* expression) is reported from 2 days post fertilization (dpf), the entrance of mature oligodendrocytes in the retina does not occur until 12 dpf (Brösamle & Halpern, 2002; Buckley et al., 2008). To complicate this story, typical oligodendrocyte markers like Olig2 and Sox10 are present in other cells such as neurons (Sagner et al., 2018) and regions outside the CNS (Santos‐Ledo et al., 2017). More interestingly, OPCs produce other members from the Sox family. For example, Sox2 has been implicated in the development of the visual system (Graham et al., 2003; Mercurio et al., 2019), controlling proliferation and cell fate. While Sox10 remains in mature oligodendrocyte, Sox2 only remains in some oligodendrocytes and neurons. Its function in differentiated cells is still a matter of debate (DeOliveira‐Mello et al., 2019).

This work intends to clarify the developmental origin and specification of OPCs in the zebrafish visual system, defining the characteristics of these cells, as well as the onset of myelination of visual tracts. Using the transgenic line *Tg(olig2:EGFP)*, we show the origin of the OPCs that will colonize the ON in the POA of the hypothalamus at 2 dpf, and how they penetrate the ON from the optic chiasm. These OPCs present round morphologies, few processes, and are positive for Sox2. As they invade and ensheath the ON at 5 dpf, they also become positive for *sox10:tagRFP* and present more projections. By 7 dpf, fully differentiated oligodendrocytes only colocalize with *sox10:tagRFP*. We also show other *olig2:EGFP/sox10:tagRFP* oligodendrocytes in the mesencephalon that differentiate earlier than those in the ON. Our results also indicate that Olig2 and Sox2 are involved in the differentiation of other retinal glial types such as Müller cells. Finally, to analyze early myelination, we used the *tg(mbpa:tagRFPt)* line and we found that *olig2:EGFP* become positive for *mbpa:tagRFPt* at 5 dpf in the ON chiasm. This reveals that the zebrafish OPCs that myelinate the ON have a similar origin to other species.

## 2 MATERIAL AND METHODS

### 2.1 Animals

Zebrafish embryos were obtained by natural mattings. Eggs were raised in E3 medium at 28.5°C, and collected at different stages, according to Kimmel et al. (1995). We employed several transgenic lines: *Tg(olig2:EGFP*; ZDB‐TGCONSTRCT‐070117‐167) (Shin et al., 2003), *Tg(sox10:tagRFP*; ZDB‐TGCONSTRCT‐150316‐1) (Blasky et al., 2014), and *Tg(mbpa:tagRFPt*; ZDB‐TGCONSTRCT‐190408‐2) (Ravanelli et al., 2018). All lines were kindly donated by Bruce Appel. In order to perform colocalization analysis, double transgenic lines were also bred: *Tg(olig2:EGFP*;*sox10:tagRFP*) and *Tg(olig2:EGFP*;*mbpa:tagRFPt*). All specimens were deeply anesthetized in tricaine methane sulfonate before sacrifice, according to Spanish and European laws (2010/63/EU; RD 53/2013; Ley 32/2007; and OrdenECC/566/2015).

All protocols were performed according to the European Union Directive 86/609/EEC and Recommendation 2007/526/EC, regarding the protection of animals used for experimental and other scientific purposes, enforced in Spanish legislation under the law 6/2013. All protocols were approved by the Bioethics Committee of the University of Salamanca.

For each stage and staining, at least 10 embryos from three different parents were used.

### 2.2 Embryo manipulation

For tissue sections, embryos/larvae were hand‐dechorionated when necessary and fixed in 4% paraformaldehyde in 0.1 M pH 7.4 phosphate buffer saline overnight at 4°C. After three washes in PBS, embryos were embedded in a solution containing 10% sucrose and 1.5% agarose. Blocks were cryoprotected in 30% sucrose in PBS overnight at 4°C. A total of 12 μm coronal sections were obtained in a cryostat (Thermo Scientific HM560).

For life imaging, embryos at the proper stage were anesthetized as usual and embedded in 1.5% low melting agarose (ThermoFisher Scientific R0801) in E3 medium.

### 2.3 Immunohistochemistry

Sections were washed several times in PBS and incubated for 90 min in 5% normal donkey (DK) serum in PBS with 0.2% Triton X‐100 at room temperature. After that, primary antibodies (Table 1) were incubated overnight in 5% normal DK serum, 0.2% Triton X‐100, and 1% dimethyl sulfoxide at 4°C. Sections were washed in PBS and then incubated 90 min at RT in darkness with a 1:400 dilution of Alexa 488, Alexa 555, or Alexa 647 fluorescent secondary antibodies (Table 2), in a buffer containing 5% normal DK serum in PBS. Next, sections were washed in PBS and then incubated for 10 min in 1:10000 4′, 6‐diamidino‐2‐fenilindol (DAPI; Sigma) for nuclei counterstaining. Sections were washed thoroughly and mounted with Fluoromount‐G® Mounting Medium (Invitrogen). Since we observed some quenching of the GFP fluorescence in the *tg(olig2:EGFP)*, we also used an anti‐GFP staining in sections. The *tg(sox10:tagRFP)* was robust enough and we never used an antibody.

**TABLE 1:** Primary antibodies

Antigen	Host	Source	Dilution	Observation
GFP	Goat	Abcam; ab5450	1:1000	Reinforce GFP
Sox2	Rabbit	Abcam; ab97959	1:500	Transcription factor
Calretinin (CR)	Mouse	Swant; 6b3	1:1000	Calcium‐binding protein
Glial fibrillary acid protein (GFAP)	Mouse	Sigma Aldrich; G6171	1:300	Typical astrocyte cytoskeleton protein
Glutamine synthetase (GS)	Mouse	Millipore; mab302	1:500	Glutamine catabolism enzyme
Pax2	Rabbit	Covance; PRB‐276P	1:500	Transcription factor

**TABLE 2:** Secondary antibodies

Antigen	Source	Conjugated	Dilution
Anti‐goat	Jackson Inmuno Research	Alexa 488	1:400
Anti‐rabbit	Jackson Inmuno Research	Alexa 555	1:400
Anti‐mouse	Jackson Inmuno Research	Alexa 647	1:400

### 2.4 Image acquisition

All images were obtained with a *LeicaStellaris* (inverted DMI8) microscope. Living embryos *tg(olig2:EGFP;mbpa:tagRFPt)* were imaged using a 20× objective from the dorsal side. Sections were imaged using a 40× oil immersion objective. In the case of the sections, four tiles were acquired and automatically assembled by the software. Acquired z‐stacks were transformed into maximum intensity projections using the LAS X software from Leica. Images were later cropped or rotated in ImageJ. Bright and contrast were only adjusted for better visualization. Finally, figures were built using *Photoshop CS5*.

### 2.5 Quantification and statistics

Colocalization of *tg(olig2:EGFP; mbpa:tagRFPt)* was quantified using the manual Cell Counter plugin included in (Fiji is Just) ImageJ. Six embryos were used per stage. First, the total number of *mbpa:tagRFPt* cells in the ON chiasm area was counted. Then, we also quantified the number of *olig2:EGFP* cells in this region and analyzed how many of them presented a total or partially overlapping *mbpa:tagRFPt*. ANOVA test with a Bonferroni post‐test statistics was performed using Graphpad Prism.

## 3 RESULTS

### 3.1 OPCs markers are detected from 2 dpf onwards

OPCs have been studied for a long time and their role in health and disease has been pointed out by several groups (Clayton & Tesar, 2021). However, we lack a full picture of their maturation and the beginning of myelination. Thus, to identify OPCs, we used the transgenic line *tg(olig2:EGFP)*. Our protocol quenched the EGFP fluorescence, so we amplified it by immunohistochemistry. Then, to characterize these *olig2:EGFP* cells we detected two transcription factors that have been implicated in OPCs differentiation (Takada & Appel, 2010; Takada et al., 2010): Sox2 by immunohistochemistry and Sox10 by breeding the double transgenic line *tg(olig2:EGFP; sox10:tagRFP)*.

**FIGURE 1:**
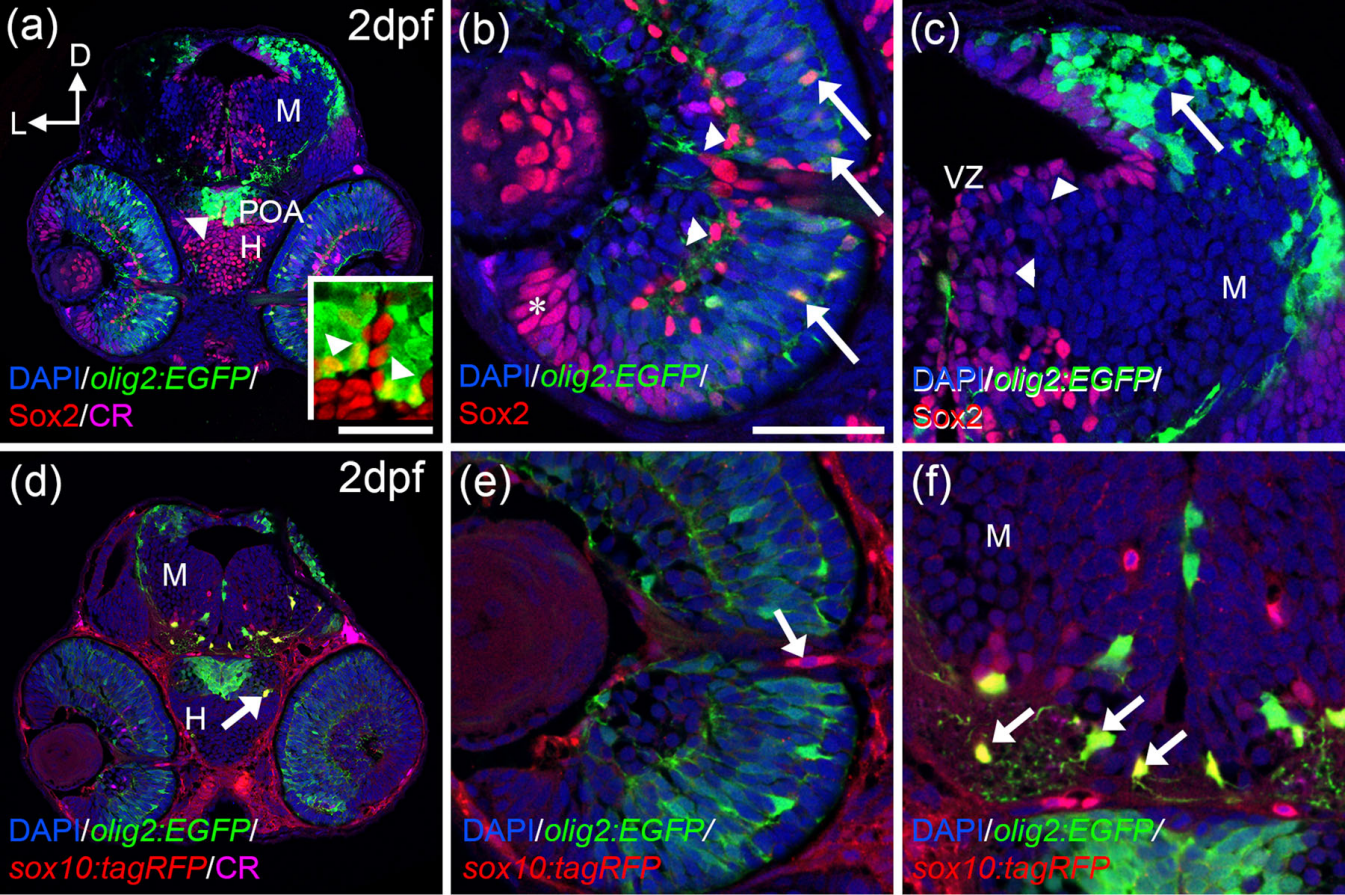
OPCs markers are detected from 2 dpf onwards. Distribution of *olig2:EGFP*/Sox2 (a–c) and *olig2:EGFP/sox10:tagRFP* (d–f) cells. *olig2:EGFP* and Sox2colocalize in the POA (arrowheads in a and inset in a), central outer retina (arrows in b), but not in the inner (arrowheads in c) or peripheral retina (asterisk in c). *Olig2:EGFP* cells are located in the dorsal optic tectum (arrow in c) but do not colocalize with Sox2 that is present in the VZ (arrowhead in c). *sox10:tagRFP* is absent from the retina except cells around the ON (d, arrow in e). In the hypothalamus and optic tectum, arborized *olig2:EGFP* cells also present *sox10:tagRFP* (arrows in d and f). Calretinin (CR) is present in several neurons, such as ganglion cells and is used to label the ON. D: dorsal; H: hypothalamus; L: lateral; M: mesencephalon; ON: optic nerve; POA: preoptic area; VZ: ventricular zone. Scale bar in a, d: 100 μm; in b, c, e, f: 50 μm

*Olig2:EGFP* was present from early somitogenesis, but the first colocalization events with Sox2 and *sox10:tagRFP* were detected at 2 dpf (Figure 1). At this stage in the visual system, we observed *olig2:EGFP* cells in the retina, the POA, and the ventral and dorsal mesencephalon (Figure 1a,d). The central part of the retina contained elongated *olig2:EGFP* that colocalized with Sox2 (Figure 1a,b). Their position, morphology, and the characterization that we performed at later stages (Figure 3) would suggest that they are differentiating glial cells. Other Sox2 cells (negative for *olig2:EGFP*) were clustered in the peripheral retina and more dispersedly in the central part (Figure 1a,b). In the mesencephalon, *olig2:EGFP* did not colocalize with Sox2, that was restricted to the ventricular zone (Figure 1a,c). Interestingly, *olig2:EGFP* cells in the dorsal POA close to the midline colocalized with Sox2 (Figure 1a and inset). Based on the evidence in other models (Bribián et al., 2006; Ono et al., 2017) and their position, we identify these *olig2:EGFP*/Sox2 cells as the first OPCs that will invade the ON.

Since we detected many *olig2:EGFP* cells negative for Sox2 in the mesencephalon, we wondered if they presented other members of this family. Sox10 is an important transcription factor during oligodendrocyte differentiation (Krasnow et al., 2018; Modzelewska et al., 2016). So the colocalization between Olig2 and Sox10 was analyzed using the double transgenic line *tg(olig2:EGFP; sox10:tagRFP). sox10:tagRFP* was absent in the retina except from some cells in the periphery of the optic nerve head (ONH) (Figure 1d,e). The *olig2:EGFP* cells in the inner hypothalamus and OT were positive for *sox10:tagRFP* (Figure 1d,f). Based on their position, far from the ventricular proliferative zones but close to the POA, and their arborized morphology, we identified these cells as OPCs differentiating into oligodendrocytes. As expected, we also observed single labeled *sox10:tagRFP* cells in the mesencephalon since this transcription factor is expressed in cells other than oligodendrocytes (Figure 1f) (Santos‐Ledo et al., 2017).

### 3.2 From 3 dpf onwards *olig2:EGFP* cells spread throughout the visual system

**FIGURE 2:**
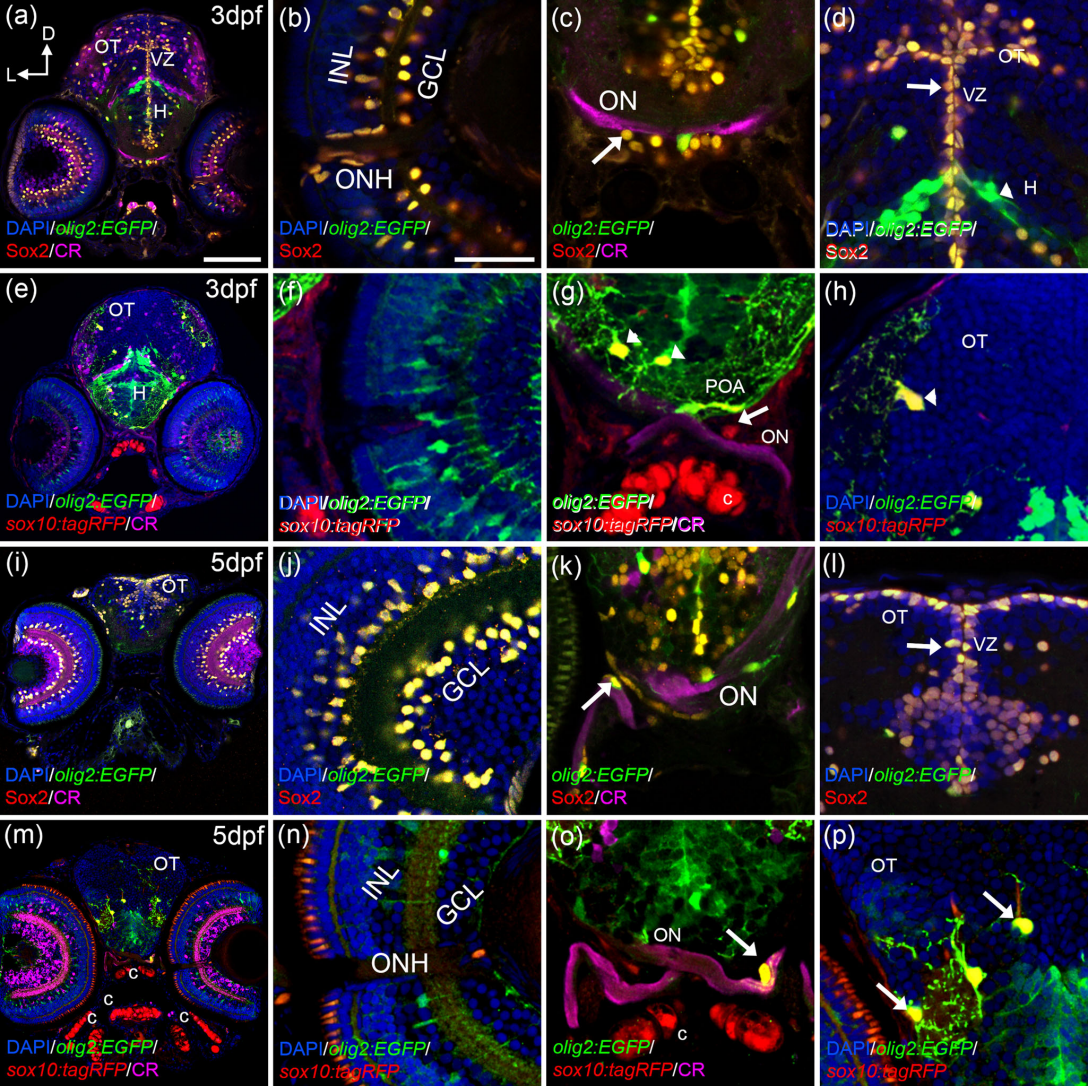
From 3 dpf onwards, OPCs cells spread throughout the visual system. At 3 dpf (a–h), *olig2:EGFP*/Sox2 cells are present in the INL, GCL, ONH (a, b), the optic chiasm (arrow in c), and the ventricular zones of the brain (arrow in d). Arborized *olig2:EGFP* cells are negative for Sox2 (arrowhead in d). *sox10:tagRFP* cells are present in the ONH (e, f) and the optic chiasm (e, arrow in g). Arborized *olig2:EGFP*/*sox10:tagRFP* cells are located in the POA with their projections surrounding the ON (arrowheads in g). OT also presented *olig2:EGFP*/*sox10:tagRFP* cells (arrowhead in h). At 5 dpf (i–p), *olig2:EGFP*/Sox2 cells are present in the INL, GCL (i, j), ON (arrow in k), and ventricular zones (arrow in l). *olig2:EGFP*/*sox10:tagRFP* cells are found in the ON with projections surrounding it (m, arrow in o), arborized *olig2:EGFP*/*sox10:tagRFP* cells are also present in the OT (arrows in p). Calretinin (CR) labels the ganglion cells and the ON. C: cartilage; D: dorsal; GCL: ganglion cell layer; H: hypothalamus; INL: inner nuclear layer; L: lateral; ON: optic nerve; ONH: optic nerve head; OT: optic tectum; POA: preoptic area; VZ: ventricular zone. Scale bar in a, e, i, m: 100 μm; in b, c, d, f, g, h, j, k, l, n, o, p: 50 μm

At 3 dpf, when retinal lamination is evident in zebrafish, *olig2:EGFP*/Sox2 cells were observed in the inner nuclear layer and in the ganglion cell layer (Figure 2a,b). *olig2:EGFP*/Sox2 cells were also detected in the periphery of the ONH (Figure 2b). Based on their position, these cells could be interneurons and retinal glial cells. *olig2:EGFP*/Sox2 cells were very abundant in the VZ of the optic tectum and hypothalamus (Figure 2a,d). We also found round *olig2:EGFP*/Sox2 around the prechiasmatic region of the ON (labeled with the marker of ganglion cells calretinin, CR) (Figure 2c). Based on the double labeling and their position, we considered these cells as the first OPCs associated to the ON.

At 3 dpf, none of the retinal *olig2:EGFP* cells show *sox10:tag*RFP (Figure 2e,f). In the OT and the hypothalamus, arborized *olig2:EGFP* cells colocalized with *sox10:tagRFP* (Figure 2e,g,h). Interestingly, the *olig2:EGFP/sox10:tagRFP* cells closest to the ON extended their projections toward the postchiasmatic region of the ON (Figure 2g). However, we did not observe any evidence of myelination onset until 5 dpf (Figure 4). Based on their more arborized morphology and the presence of *sox10:tagRFP*, we consider these cells as differentiating oligodendrocytes.

At 5 dpf, we observed little changes respect to 3 dpf in the retina and OT. *olig2:EGFP* colocalized with Sox2 in the INL, GCL, and VZ of the OT (Figure 2i–k). Arborized oligodendrocytes in the OT and hypothalamus presented both *olig2:EGFP* and *sox10:tagRFP* (Figure 2m,n,p) and were negative for Sox2 (described later in Figure 5). The most striking change was found in the ON, *olig2:EGFP/sox10:tagRFP* cells around the ON showed projections colocalizing with CR suggesting that they are differentiated oligodendrocytes and the ensheathing process is starting (Figure 2o).

### 3.3 Olig2 is also involved in the differentiation of other glial cells in the retina

**FIGURE 3:**
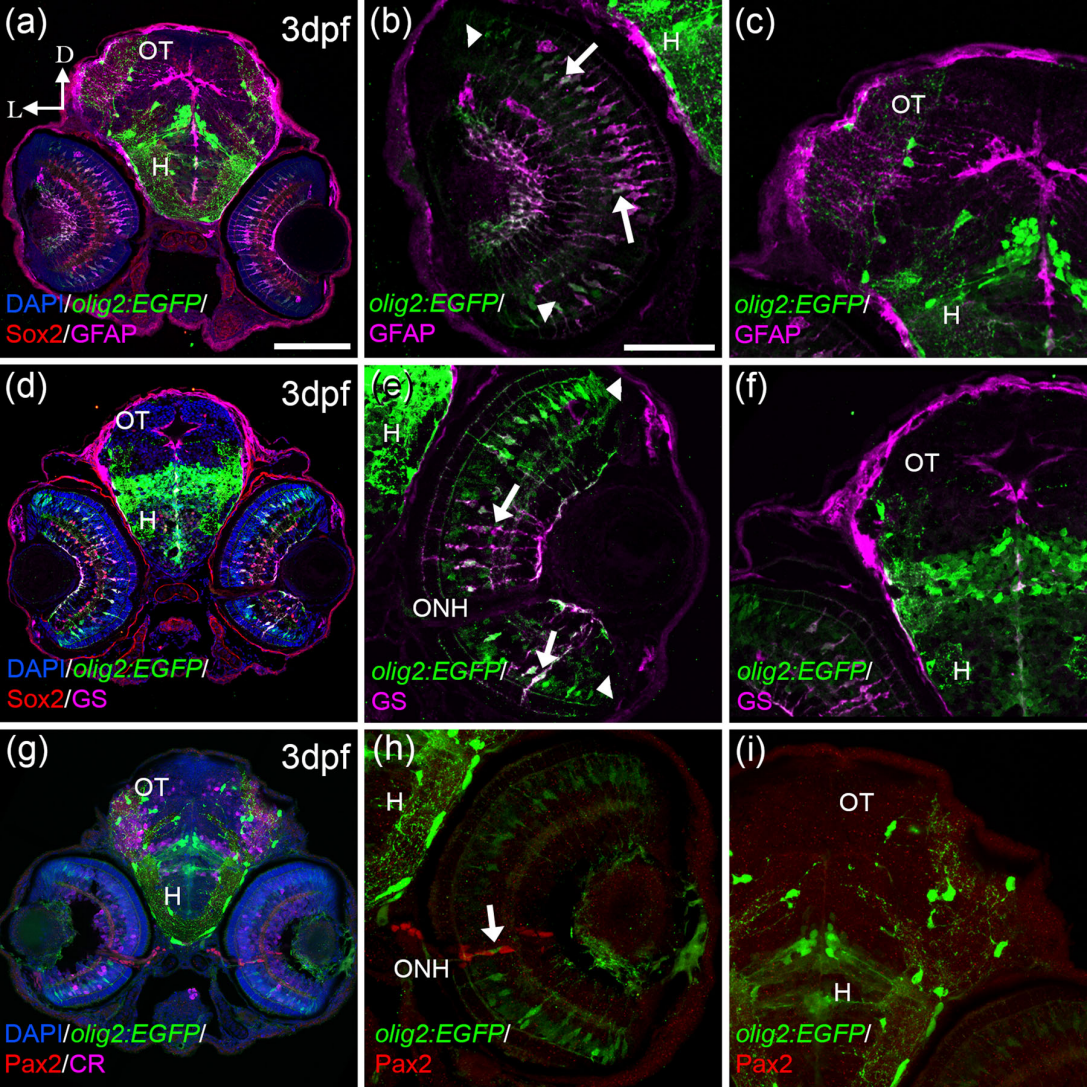
*olig2:EGFP* cells present other glial markers in the retina; GFAP and GS. *olig2:EGFP* cells also colocalized with Müller markers: GFAP (a, b) and GS (d, e) in the retina but not in the OT (c, f). Colocalization was detected mostly in the central area of the retina (arrows in band e), while cells in the periphery were just *olig2:EGFP* (arrowheads in b and e). *olig2:EGFP* cells in the periphery of the ONH do not colocalize with Pax2, typical maker for reticular astrocytes (g–i), although these two populations are very close (arrow in h). D: dorsal; L: lateral; H: hypothalamus; ONH: optic nerve head; OT: optic tectum. Scale bar in a, d, g: 100 μm; in b, c, e, f, h, i: 50 μm

Our data indicated that OPCs (*olig2:EGFP*/Sox2 cells) originated in the POA and then extended toward the ON. However, we also observed this colocalization in the retina (Figure 1 and 2). The *olig2:EGFP*/Sox2 cells in the INL presented elongated morphologies characteristic of Múller glia (Figure 1 and 2), which are very important for the retinal maintenance and structure (Thummel et al., 2008). Olig2 have been implicated in the differentiation of other glial cells (Cai et al., 2007). Thus, we used typical markers for Müller cells (GFAP and GS) (Santos‐Ledo et al., 2011). Indeed, *olig2:E*GFP cells in the central part of the retina, the most differentiated region, colocalized with GFAP (Figure 3a,b) and GS (Figure 3d,e). The previously identified arborized oligodendrocytes in the OT (Figure 1 and 2) did not colocalize with GFAP (Figure 3c) or GS (Figure 3f). The retina also contains reticular astrocytes which are very important during the formation and the regeneration of the ON (Parrilla et al., 2013). Since we found *olig2:EGFP* cells in this area (Figure 2b), we wondered if they colocalized with Pax2, the typical marker for these astrocytes. They did not but they were close to the Pax2+ astrocytes (Figure 3g–i).

### 3.4 *Olig2:EGFP* cells are observed differentiated from 7 dpf in the ON

**FIGURE 4:**
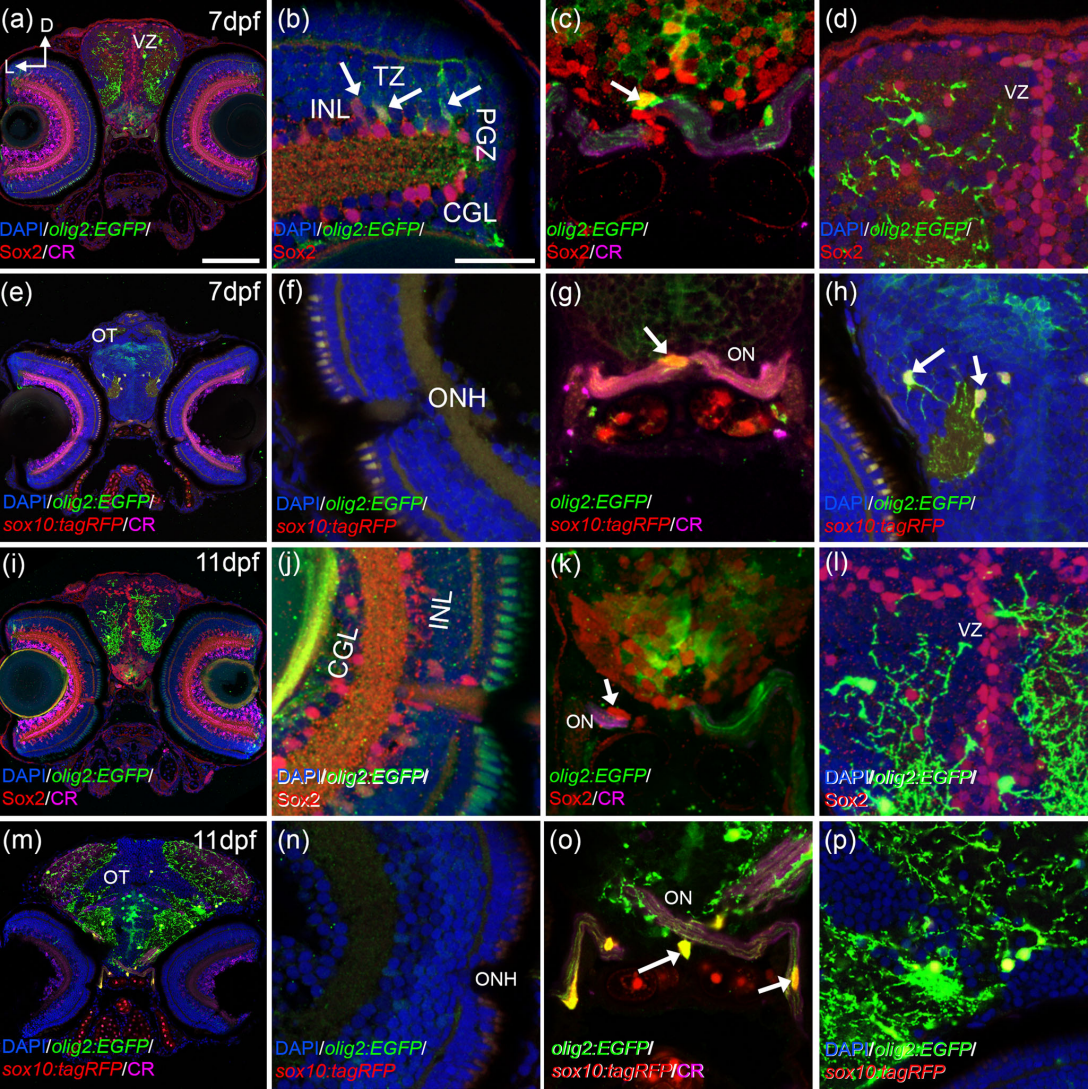
*olig2:EGFP* cells are differentiated from 7 dpf. In the retina at 7 dpf, *olig2:EGFP* cells are restricted to the PGZ and the TZ (a, b). But only the cells in the TZ colocalize with Sox2 (arrows in b). Sox2 that do not show *olig2:EGFP* are abundant in the proliferative areas of the brain (c, d). *olig2:EGFP* occasionally colocalize with Sox2 in the optic nerve chiasm at 7 dpf (arrow in c). At 7 dpf *olig2:EGFP/sox10:tagRFP* cells were found in the ON chiasm (e, arrow in g) and in other parts of the brain (arrows in h). Differentiated part of the retina was empty of *olig2:EGFP/sox10:tagRFP* cells (f). At 11 dpf, Sox2 keeps its pattern in the retina (i, j) and in the ON (arrow k). In the OT, Sox2 cells are restricted to the ventricular area (l). *olig2:EGFP/sox10:tagRFP* cells are abundant in the ON with obvious projections (m, arrows in o) and there are none in the retina (n). Oligodendrocytes in the OT are *olig2:EGFP/sox10:tagRFP* (p). Calretinin (CR) is used to label ganglionar cells and ON. D: dorsal; GCL: ganglion cell layer; INL: inner nuclear layer; L: lateral; ONH: optic nerve head; ON; optic nerve; OT: optic tectum; PGZ: proliferative germinal zone; VZ: ventricular zone. Scale bar in a, e, i, m: 100 μm; in b, c, d, f, g, h, j, k, l, n, o, p: 50 μm

The colocalization of *olig2:EG*FP and Sox2 changed drastically at 7 dpf (Figure 4) respect to 5 dpf (Figure 3). In the retina, *olig2:EG*FP cells were located in the proliferative germinal zone, where retinal progenitors are, and did not colocalize with Sox2 (Figure 4a,b). However, *Olig2:EGFP* cells in the transition zone close to the PGZ, where maturing cells are located, colocalized with Sox2 (Figure 4a,b). We did not observe colocalization in the OT (Figure 4d). In the ON, we found *olig2:EGFP* cells with little arborization that colocalized with Sox2 (Figure 4c). We identified these *olig2:EGFP*/Sox2 cells as OPCs contributing to the development of the ON. We also detected Sox2 cells with no *olig2:EGFP* closely associated with the ON (Figure 4c).

At 7 dpf, *olig2:EGFP/Sox10:tagRFP* cells maintained its distribution compared to 5 dpf. No colocalization in the retina (Figure 4e,f) but arborized *olig2:EGFP/Sox10:tagRFP* cells in the OT (Figure 4h) and in the ON where they showed more projections (Figure 4g). We identified these double‐labeled cells with long projection as differentiated oligodendrocytes.

At 11 dpf, the pattern of *olig2:EGFP*/Sox2 cells was identical to 7 dpf (Figure 4i,j,l). Sox2 colocalized very rarely with *olig2:EGFP* in the ON (Figure 4k). In relation to the differentiated oligodendrocytes (*olig2:EGFP/sox10:tagRFP*), the most important difference compared to 7 dpf is the increased abundance of these cells in the ON and OT with greater arborization (Figure 4 m–p).

### 3.5 Oligodendrocytes switch from Sox2 to *sox10:tagRFP* at 5 dpf in the ON

Our data pointed to an abundance of *olig2:EGFP/Sox2* cells at 3 dpf in the ON (Figure 3). As the embryo matured, we observed a reduction in these cells but an increase in arborized *olig2:EGFP/sox10:tagRFP* (Figure 4). To clarify if these cells were the same cells changing their profile, we wondered if they ever presented Sox2 and *sox10:tagRFP* at the same time.

**FIGURE 5:**
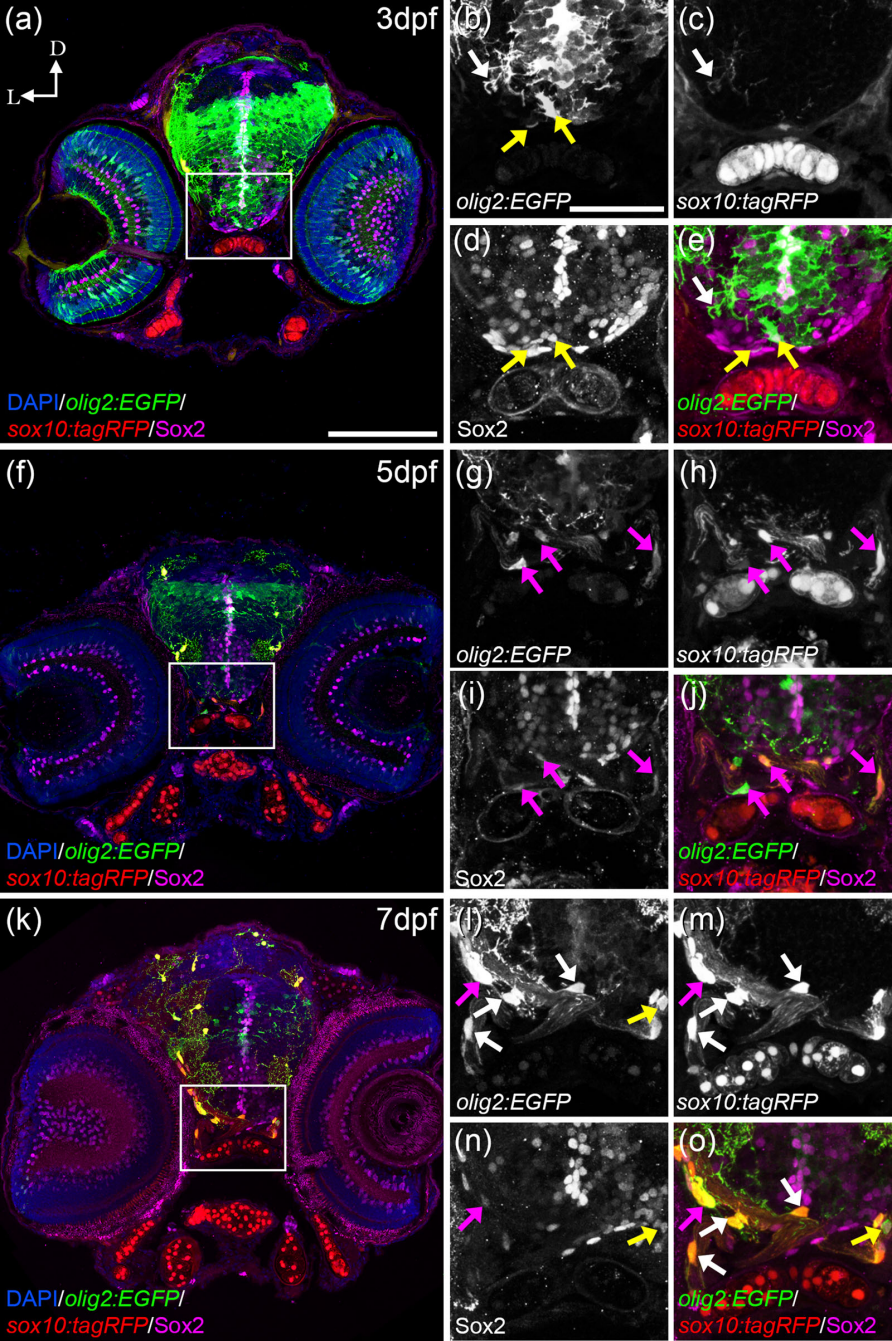
*olig2:EGFP* cells switch from Sox2 to *sox10:tagRFP*. At 3 dfp (a), *olig2:EGFP* cells with round morphologies and slightly arborized in the postchiasmatic ON colocalized with Sox2 but not with *sox10:tagRFP* (a, yellow arrows in b, d, and e). Fully arborized *olig2:EGFP* cells in the ventral OT colocalized with *sox10:tagRFP* and not with Sox2 (a, white arrows in b, c, and e). At 5 dpf (f), *olig2:EGFP* with elongated morphologies and arborization colocalize simultaneously with Sox2 and *sox10:tagRFP* (f, magenta arrows in g–j). At 7 dfp (k), arborized *olig2:EGFP* cells that present many projections colocalize only with *sox10:tagRFP* (k, white arrows in l, m, and o); while some *olig2:EGFP* cells with less arborization colocalize with Sox2 and *sox10:tagRFP* (magenta arrows in l–o) and those with round morphologies colocalized only with Sox2 and not *sox10:tagRFP* (yellow arrows in l, n, and o). D: dorsal; L: lateral. Scale bar in a, f, k: 100 μm; in b, c, d, e, g, h, I, j, l, m, n, o: 50 μm

At 3 dpf in the ON region, *olig2:EGFP* cells with round morphologies or slightly arborized colocalized with Sox2 but not with *sox10:tagRFP* (Figure 5a, yellow arrows in b, d, and e). More arborized oligodendrocytes in the ventral OT presented *sox10:tagRFP* but no Sox2 (Figure 5a, white arrows in b, c, and e). Interestingly, at 5 dpf *olig2:EGFP* cells in the ON, with elongated morphologies and short projections, colocalized with Sox2 and *sox10:tagRFP* simultaneously (Figure 5f, magenta arrows in g–j). At 7 dpf, round *olig2:EGFP* with almost no arborization colocalized only with Sox2 (Figure 5k, yellow arrows in l, n, o); *olig2:EGFP* cells with round morphologies and more arborization colocalized with *sox10:tagRFP* and Sox2 (Figure 5k, magenta arrows in l–o); and, *olig2:EGFP* with many protections that wrap the ON colocalized only with *sox10:tagRFP* (Figure 5k, white arrows in l, m, and o). This would suggest that OPCs (*olig2:EGFP*) present Sox2, they migrate to their final positions where they switch to Sox10 as they differentiate. In case of the zebrafish ON, this transition first occurs at 5 dpf.

### 3.6 The first evidence of the ON myelination is detected at 5 dpf

An important function of oligodendrocytes is the myelination of axons within the CNS. To follow up the relation between Olig2 and the myelination process, we established a double transgenic line(*olig2:EGFP/mbpa:tagRFPt*) and analyze the distribution of the transgenes in vivo at 5, 7, and 11 dpf. Mbpa protein binds to myelin and thus can be useful to understand when myelination is occurring (Hughes & Appel, 2020).

**FIGURE 6:**
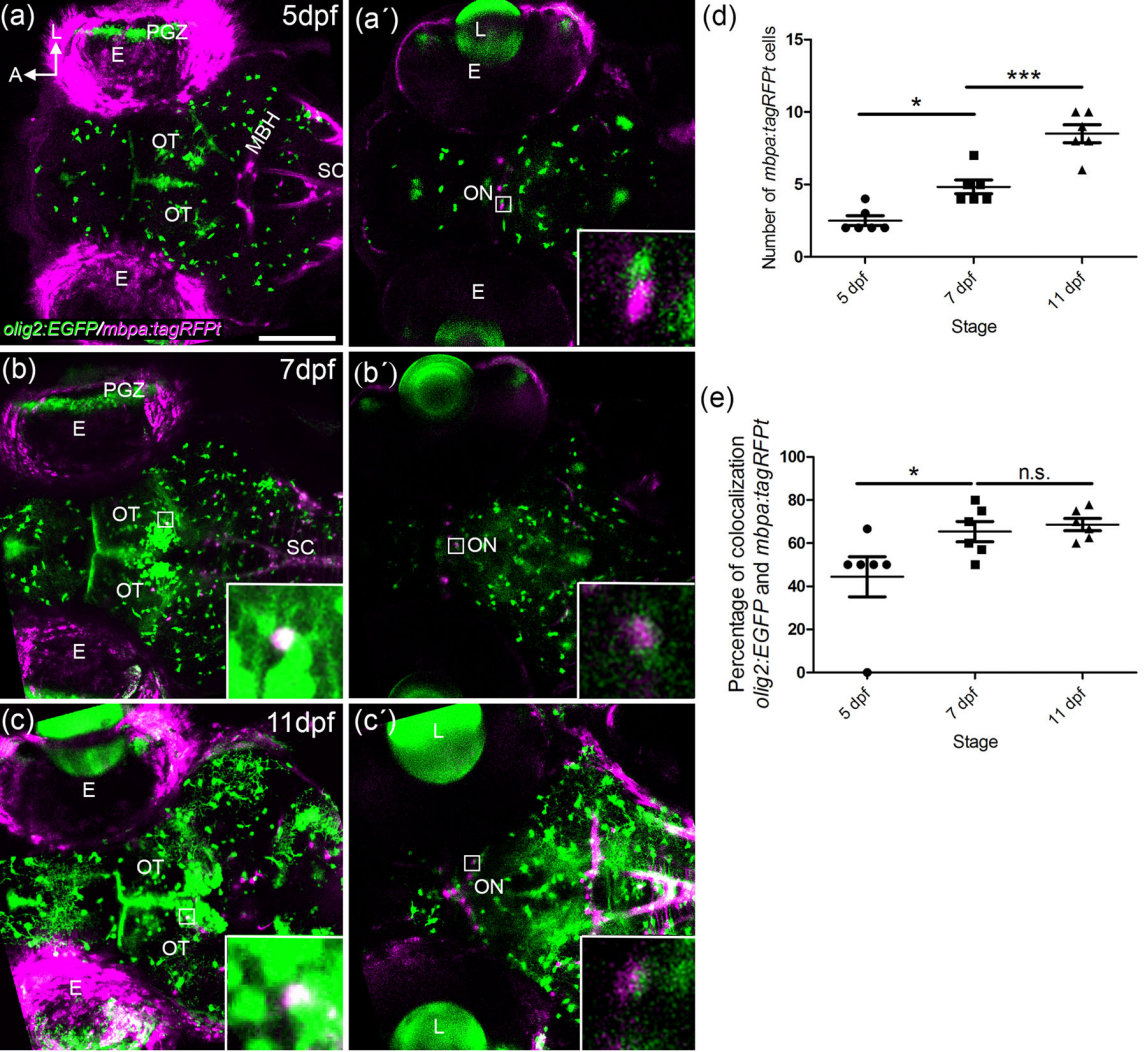
Myelination of the visual system. Confocal images of whole mount *olig2:EGFP/mbpa:tagRFPt* embryos at 5 (a, a′), 7 (b, b′), and 11 (c′) dpf. A 25 μm thick stack is shown as max intensity projection in the OT area (a, b, and c) and the optic nerve chiasm (a′, b′, and c′). Detection of *mbpa:tagRFPt* starts at 5 dpf in the ON (a′). The number of *mbpa:tagRFPt* cells increase significantly from 3–4 cells at 5 dpf to 8–10 cells at 11 dpf (a′, b′, c′, quantified in d). A total of 40% *olig2:EGFP* colocalize with *mbpa:tagRFPt* at 5 dpf. This percentage increases to 60% from 5 dpf to 7 dpf (quantified in e). E: eye: L: lens; MBH; midbrain hindbrain boundary; ON: optic nerve; OT: optic tectum; PGZ: proliferative germinal zone; SC: spinal cord. Scale bar: 100 μm

The first *mbpa:tagRFPt* cells of the ON were detected at 5 dpf in the optic chiasm area (Figure 6a,a′). We counted 2 or 3 *mbpa:tagRFPt* cells per embryo (quantified in figure 6d). This number increased to 5 cells at 7 dpf (Figure 6b′) and to 8–10 cells at 11 dpf (Figure 6c′; quantified in Figure 6d).

We previously identified key morphological changes of the *olig2:EGFP* cells starting at 5 dpf. These included cellular elongation and increased arborization. We wondered if this timeline was coincidental with the detection of *mbpa:tagRFPt*. *olig2:EGFP* cells in the OT with full arborization colocalized with *mbpa:tagRFPt* (insets in Figure 6b,c). Interestingly, in the ON, only 40% of *olig2:EGFP* cells showed this colocalization (inset in Figure 6a′, quantified in Figure 6e). These colocalization events significantly increased to more than 60% at 7 dpf (inset in Figure 6b′ and quantified in Figure 6e). We observed no further changes between 7 and 11 dpf in relation to the colocalization (inset in Figure 6c′ and quantified in Figure 6e). These data would again suggest that crucial changes are occurring between 5 and 7 dpf.

## 4 DISCUSSION

Our analysis show that the visual OPCs (*olig2:EGFP*/Sox2) are located in the POA at 2 dpf. Olig2 is a key transcription factor during oligodendrocyte differentiation (Zou & Hu, 2021) and Sox2 maintain stem cell properties (Wegner & Stolt, 2005). At 3 dpf, we can find *olig2:EGFP*/Sox2 cells also in the chiasmatic region of the ON where they start acquiring a more elongated morphology. Five dpf is a key stage since we have observed triple colocalization events (*olig2:EGFP*/Sox2/*sox10:tag*RFP) in the ON and increased cellular arborization. Since these cells maintain Sox2 but also colocalize with *sox10:tag*RFP, a mature oligodendrocytic marker (Parrilla et al., 2016), we identified them as differentiating oligodendrocytes. Finally, from 7 dpf onwards, most of the *olig2:EGFP* cells in the ON colocalize with *sox10:tagRFP* but not with Sox2. We have identified these cells as fully differentiated oligodendrocytes. From 7 dpf, the three different populations (*olig2:EGFP*/Sox2;*olig2:EGFP*/Sox2/*sox10:tagRFP*; *olig2:EGFP*/*sox10:tagRFP*) coexist in the ON.

Tian et al. (2016) suggest an extraocular origin of the oligodendrocytes that envelop the ON but start their description at 7 dpf. Other studies that use ablation or demyelination experiments also describe the formation of oligodendrocytes and their differentiation, but they do not pinpoint their origin (Chung et al., 2013; Fang et al., 2014). Our evidence supports the extraocular origin hypothesis, but indicate that the first OPCs (*olig2:EGFP*/Sox2) originate in the POA at 2 dpf and then move to the ON arriving to the optic chiasm at 3 dpf. These OPCs will then colonize pre and postchiasmatic regions. Since we do not detect evidence of myelin‐related proteins until 5 dpf, this would suggest an earlier contribution of these cells to the development of the ON where they could have more functions besides myelination. In fact, very recently, Xiao et al. (2022) show that OPCs also contribute to axon remodeling during zebrafish ON development as early as 5 dpf.

The timeline described by us (graphical abstract) coincides with data from mice and chicken, where OPCs migrate through the brain until the optic chiasm, from where they finally colonize the rest of the ON (Bribián et al., 2006; Gao & Miller, 2006; Merchán et al., 2007). In mouse and chicken, the OPCs that populate the ON are originated in the third ventricle, but with some differences. In mouse, they are dorsal to the developing optic chiasm (Gao & Miller, 2006), while in chicken they are in the ventral midline region (Ono et al., 1997). Thus, our data suggest that OPCs in zebrafish origin is closer to mouse. Transgenic lines, like those used by us, have some limitations since they might not be labeling oligodendrocytes at all stages and cells might retain GFP after the expression of the promotor is stopped. However, since in zebrafish we lack a specific marker of oligodendrocytes, they allow linage tracing and provide strong and useful information (Ravanelli et al., 2018; Xiao et al., 2022).

We have also found *olig2:EGFP* cells in the retina. At early stages (2 to 5 dpf) together with Sox2, a progenitor marker (Bylund et al., 2003) and later restricted to the proliferative growth zone where retinal progenitors are (Arenzana et al., 2011). At 3 dpf, *olig2:EGFP* cells also present markers for Müller glia (GS+). This expression has been associated with developmental and regenerative processes since Müller cells act as retinal neural progenitors (Fimbel et al., 2007; Nakamura et al., 2006; Shibasaki et al., 2007; Thummel et al., 2008). Surrounding the ONH, we have found also *olig2:EGFP*/Sox2 cells at 3 dpf that lose the *olig2:EGFP* expression by 5 dpf. Based on our previous experience and since Olig2 is involved in the differentiation of other glial cells (Cai et al., 2007), we hypothesized that they could be the reticular astrocytes that form the optic stalk. However, they lack the characteristic marker Pax2 (Parrilla et al., 2012, 2013; Tiwari et al., 2014). Thus, our data indicate that Olig2 and Sox2 might play a role during the differentiation of the retinal glial cells (DeOliveira‐Mello et al., 2019). To unravel the specific role of these transcription factors would require further experiments.

While the onset of myelination in the spinal cord has been deeply studied (Hines, 2021; Kordes et al., 2005; Takada & Appel, 2010), there is not much known regarding the myelination in the zebrafish visual system. To study the relationship between oligodendrocytes and the first steps of myelination in vivo, we used the transgenic line *mbpa:tagRFPt* that allow us to observe the activation of *myelin binding protein a* gene (Hughes & Appel, 2020; Xiao et al., 2022). In zebrafish CNS, the Mauthner axons are the first ones to become myelinated by 3 dpf (Buckley et al., 2008). *Mbpa*, and other genes related to myelination, are clearly expressed in some brain regions by 4 dpf (Bai et al., 2011; Brösamle & Halpern, 2002; Jung et al., 2010; Pinzon‐Olejua et al., 2017). These authors do not find myelinated axons in the ON by electron microscopy until 7 dpf (Brösamle & Halpern, 2002). However, we detected the first *olig2:EGFP/mbpa:tagRFPt* cells in the ON at 5 dpf. All together this data indicate that myelination is a progressive process that might be triggered in different places simultaneously and that there is a lag between the expression of myelin‐related proteins and myelination. In fact, it has been shown that OPCs undergo complex morphological changes over time in response to neural activity before myelination can occur (Krasnow et al., 2018). Our results match with these data. The first projections from *olig2:EGFP*/Sox2/*sox10:tagRFP* cells that envelop the ON are detected at 5 dpf, as well as the first *mbpa:tagRFPt*. By 11 dpf, projections are becoming sheaths that envelope the ON (labeled with CR) and *mbpa:tagRFPt* cells increase significantly. We did not find a full colocalization between *mbpa:tagRFPt* and *olig2:EFGP* transgenes, but a close relationship between them. As previously suggested by Hughes and Appel (2020) using the same transgenic line, we are detecting processes associated with myelin rather than oligodendrocytes bodies. Concomitantly to the detection of *mbpa:tagRFPt* and to the morphological changes, *olig2:EGFP* cells switch from Sox2 to *sox10:tagRFP*. This transition is obvious in the ON between 5 and 7 dpf. This might be a consequence of the transition from the proliferative state of OPCs to more mature oligodendrocytes (Ono et al., 2017). This view is reinforced by our description of the early *olig2:EGFP*/Sox2 cells in the midline, a typical neurogenic zone, that will remain just as Sox2 (Germanà et al., 2011) and by the exclusive presence of *sox10:tagRFP* in the fully differentiated oligodendrocytes in other areas such as the OT.

Our results show that OPCs in the zebrafish visual system are generated in similar brain areas to other groups of vertebrates, at earlier time points than previously described. We have also found that myelin‐related markers can be detected at 5 dpf in the optic chiasm, and that oligodendrocytes extend both dorsally and ventrally in the visual pathway. Therefore, the visual system myelination of zebrafish resembles other vertebrates despite the morphological differences and validate this model to study human diseases related to aberrant myelination. Furthermore, it would be interesting to investigate in the future whether the changes we described during development are conserved in adulthood and during regeneration. For example, oligodendrocytes expressing *sox10* have been described to be involved in mouse regeneration (Mendonça et al., 2021). Zebrafish, an animal with continuous growth and where multiple de and remyelination events occur could be an ideal model for this type of research (Chung et al., 2013; Fang et al., 2014; Zou & Hu, 2021).

## AUTHOR CONTRIBUTIONS

Adrián Santos‐Ledo and Cristina Pérez‐Montes equally contributed to this manuscript. Conceptualization: Adrián Santos‐Ledo, Cristina Pérez‐Montes, and Almudena Velasco. Methodology: Adrián Santos‐Ledo, Cristina Pérez‐Montes, and Laura DeOliveira‐Mello. Validation: Adrián Santos‐Ledo, Cristina Pérez‐Montes, and Almudena Velasco. Formal analysis: Adrián Santos‐Ledo, Cristina Pérez‐Montes, Rosario Arévalo. Investigation: Adrián Santos‐Ledo, Cristina Pérez‐Montes, Laura DeOliveira‐Mello, and Almudena Velasco. Resources: Rosario Arévalo and Almudena Velasco. Data curation: Adrián Santos‐Ledo, Cristina Pérez‐Montes, Rosario Arévalo, and Almudena Velasco. Writing—original draft: Adrián Santos‐Ledo and Almudena Velasco. Writing—review & editing: Adrián Santos‐Ledo, Cristina Pérez‐Montes, Rosario Arévalo, and Almudena Velasco. Visualization: Adrián Santos‐Ledo, Cristina Pérez‐Montes, and Almudena Velasco. Supervision: Rosario Arévalo and Almudena Velasco. Project administration: Adrián Santos‐Ledo, Rosario Arévalo, and Almudena Velasco. Funding acquisition: Almudena Velasco.

## CONFLICT OF INTEREST

The authors declare no conflict of interest that could be perceived as prejudicing the impartiality of the research reported.

### PEER REVIEW

The peer review history for this article is available at https://publons.com/publon/10.1002/cne.25440.

## Data Availability

All relevant data are within the manuscript.

## References

[cne25440-bib-0001] Arenzana, F. J. , Santos‐Ledo, A. , Porteros, A. , Aijón, J. , Velasco, A. , Lara, J. M. , & Arévalo, R. (2011). Characterisation of neuronal and glial populations of the visual system during zebrafish lifespan. International Journal of Developmental Neuroscience, 29(4), 441–449. 10.1016/j.ijdevneu.2011.02.008 21392569

[cne25440-bib-0002] Bai, Q. , Sun, M. , Stolz, D. B. , & Burton, E. A. (2011). Major isoform of zebrafish P0 is a 23.5 kDa myelin glycoprotein expressed in selected white matter tracts of the central nervous system. Journal of Comparative Neurology, 519(8), 1580–1596. 10.1002/cne.22587 21452240 PMC3903511

[cne25440-bib-0003] Baumann, N. , & Pham‐Dinh, D. (2001). Biology of oligodendrocyte and myelin in the mammalian central nervous system. Physiological Reviews, 81(2), 871–927. 10.1152/physrev.2001.81.2.871 11274346

[cne25440-bib-0004] Berry‐Brincat, A. , & Shafquat, S. (2008). Myelinated nerve fibres: A rare cause of recurrent vitreous haemorrhage. Eye, 22(1), 165–167. 10.1038/sj.eye.6703002 17992199

[cne25440-bib-0005] Blasky, A. J. , Pan, L. , Moens, C. B. , & Appel, B. (2014). Pard3 regulates contact between neural crest cells and the timing of schwann cell differentiation but is not essential for neural crest migration or myelination. Developmental Dynamics, 243(12), 1511–1523. 10.1002/dvdy.24172 25130183 PMC4237626

[cne25440-bib-0006] Bribián, A. , Barallobre, M. J. , Soussi‐Yanicostas, N. , & de Castro, F. (2006). Anosmin‐1 modulates the FGF‐2‐dependent migration of oligodendrocyte precursors in the developing optic nerve. Molecular and Cellular Neuroscience, 33(1), 2–14. 10.1016/j.mcn.2006.05.009 16876430

[cne25440-bib-0007] Brösamle, C. , & Halpern, M. E. (2002). Characterization of myelination in the developing zebrafish. Glia, 39(1), 47–57. 10.1002/glia.10088 12112375

[cne25440-bib-0008] Buckley, C. E. , Goldsmith, P. , & Franklin, R. J. (2008). Zebrafish myelination: A transparent model for remyelination? Disease Models & Mechanisms, 1(4–5), 221–228. 10.1242/dmm.001248 19093028 PMC2590821

[cne25440-bib-0009] Bylund, M. , Andersson, E. , Novitch, B. G. , & Muhr, J. (2003). Vertebrate neurogenesis is counteracted by sox1‐3 activity. Nature Neuroscience, 6(11), 1162–1168. 10.1038/nn1131 14517545

[cne25440-bib-0010] Cai, J. , Chen, Y. , Cai, W. H. , Hurlock, E. C. , Wu, H. , Kernie, S. G. , Parada, L. F. , & Lu, Q. R. (2007). A crucial role for olig2 in white matter astrocyte development. Development (Cambridge, England), 134(10), 1887–1899. 10.1242/dev.02847 17428828

[cne25440-bib-0011] Chung, A. Y. , Kim, P. S. , Kim, S. , Kim, E. , Kim, D. , Jeong, I. , Kim, H.‐K. , Ryu, J.‐H. , Kim, C.‐H. , Choi, J. , Seo, J.‐H. , & Park, H. C. (2013). Generation of demyelination models by targeted ablation of oligodendrocytes in the zebrafish CNS. Molecules and Cells, 36(1), 82–87. 10.1007/s10059-013-0087-9 23807048 PMC3887923

[cne25440-bib-0012] Clayton, B. L. L. , & Tesar, P. J. (2021). Oligodendrocyte progenitor cell fate and function in development and disease. Current Opinion in Cell Biology, 73, 35–40. 10.1016/j.ceb.2021.05.003 34153742 PMC8678156

[cne25440-bib-0013] Czopka, T. (2016). Insights into mechanisms of central nervous system myelination using zebrafish. Glia, 64(3), 333–349. 10.1002/glia.22897 26250418

[cne25440-bib-0014] DeOliveira‐Mello, L. , Lara, J. M. , Arevalo, R. , Velasco, A. , & Mack, A. F. (2019). Sox2 expression in the visual system of two teleost species. Brain Research, 1722, 146350. 10.1016/j.brainres.2019.146350 31351039

[cne25440-bib-0015] Fang, Y. , Lei, X. , Li, X. , Chen, Y. , Xu, F. , Feng, X. , Wei, S. , & Li, Y. (2014). A novel model of demyelination and remyelination in a GFP‐transgenic zebrafish. Biology Open, 4(1), 62–68. 10.1242/bio.201410736 25527642 PMC4295166

[cne25440-bib-0016] Fimbel, S. M. , Montgomery, J. E. , Burket, C. T. , & Hyde, D. R. (2007). Regeneration of inner retinal neurons after intravitreal injection of ouabain in zebrafish. Journal of Neuroscience, 27(7), 1712–1724. 10.1523/JNEUROSCI.5317-06.2007 17301179 PMC6673754

[cne25440-bib-0017] Fitz Gibbon, T. , & Nestorovski, Z. (1997). Morphological consequences of myelination in the human retina. Experimental Eye Research, 65(6), 809–819. 10.1006/exer.1997.0388 9441705

[cne25440-bib-0018] Fujita, Y. , Imagawa, T. , & Uehara, M. (2000). Comparative study of the lamina cribrosa and the pial septa in the vertebrate optic nerve and their relationship to the myelinated axons. Tissue & Cell, 32(4), 293–301. 10.1054/tice.2000.0115 11145012

[cne25440-bib-0019] Gao, L. , & Miller, R. H. (2006). Specification of optic nerve oligodendrocyte precursors by retinal ganglion cell axons. Journal of Neuroscience, 26(29), 7619–7628. 10.1523/JNEUROSCI.0855-06.2006 16855089 PMC6674293

[cne25440-bib-0020] Germanà, A. , Montalbano, G. , Guerrera, M. C. , Amato, V. , Laurà, R. , Magnoli, D. , Campo, S. , Suarez‐Fernandez, E. , Ciriaco, E. , & Vega, J. A. (2011). Developmental changes in the expression of sox2 in the zebrafish brain. Microscopy Research and Technique, 74(4), 347–354. 10.1002/jemt.20915 20734413

[cne25440-bib-0021] Graham, V. , Khudyakov, J. , Ellis, P. , & Pevny, L. (2003). SOX2 functions to maintain neural progenitor identity. Neuron, 39(5), 749–765. 10.1016/s0896-6273(03)00497-5 12948443

[cne25440-bib-0022] Hines, J. H. (2021). Evolutionary origins of the oligodendrocyte cell type and adaptive myelination. Frontiers in Neuroscience, 15, 757360. 10.3389/fnins.2021.757360 34924932 PMC8672417

[cne25440-bib-0023] Hughes, A. N. , & Appel, B. (2020). Microglia phagocytose myelin sheaths to modify developmental myelination. Nature Neuroscience, 23(9), 1055–1066. 10.1038/s41593-020-0654-2 32632287 PMC7483351

[cne25440-bib-0024] Jung, S. H. , Kim, S. , Chung, A. Y. , Kim, H. T. , So, J. H. , Ryu, J. , Park, H.‐C. , & Kim, C. H. (2010). Visualization of myelination in GFP‐transgenic zebrafish. Developmental Dynamics, 239(2), 592–597. 10.1002/dvdy.22166 19918882

[cne25440-bib-0025] Kimmel, C. B. , Ballard, W. W. , Kimmel, S. R. , Ullmann, B. , & Schilling, T. F. (1995). Stages of embryonic development of the zebrafish. Developmental Dynamics, 203(3), 253–310. 10.1002/aja.1002030302 8589427

[cne25440-bib-0026] Klionsky, D. J. , Abdel‐Aziz, A. K. , Abdelfatah, S. , Abdellatif, M. , Abdoli, A. , Abel, S. , Abeliovich, H. , Abildgaard, M. H. , Abudu, Y. P. , Acevedo‐Arozena, A. , Adamopoulos, I. E. , Adeli, K. , Adolph, T. E. , Adornetto, A. , Aflaki, E. , Agam, G. , Agarwal, A. , Aggarwal, B. B. , Agnello, M. , … Tong, C. K. (2021). Guidelines for the use and interpretation of assays for monitoring autophagy. Autophagy, 17(1), 1–382. 10.1080/15548627.2020.1797280 33634751 PMC7996087

[cne25440-bib-0027] Kordes, U. , Cheng, Y. C. , & Scotting, P. J. (2005). Sox group e gene expression distinguishes different types and maturational stages of glial cells in developing chick and mouse. Brain Research Developmental Brain Research, 157(2), 209–213. 10.1016/j.devbrainres.2005.03.009 15878625

[cne25440-bib-0028] Krasnow, A. M. , Ford, M. C. , Valdivia, L. E. , Wilson, S. W. , & Attwell, D. (2018). Regulation of developing myelin sheath elongation by oligodendrocyte calcium transients in vivo. Nature Neuroscience, 21(1), 24–28. 10.1038/s41593-017-0031-y 29230052 PMC6478117

[cne25440-bib-0029] Lillo, C. , Velasco, A. , Jimeno, D. , Cid, E. , Lara, J. M. , & Aijón, J. (2002). The glial design of a teleost optic nerve head supporting continuous growth. Journal of Histochemistry and Cytochemistry, 50(10), 1289–1302. 10.1177/002215540205001002 12364562

[cne25440-bib-0030] Lyons, D. A. , & Talbot, W. S. (2014). Glial cell development and function in zebrafish. Cold Spring Harbor Perspectives in Biology, 7(2), a020586. 10.1101/cshperspect.a020586 25395296 PMC4315925

[cne25440-bib-0031] Mathews, E. S. , & Appel, B. (2016). Oligodendrocyte differentiation. Methods in Cell Biology, 134, 69–96. 10.1016/bs.mcb.2015.12.004 27312491

[cne25440-bib-0032] Mendonça, H. R. , Villas Boas, C. O. G. , Heringer, L. D. S. , Oliveira, J. T. , & Martinez, A. M. B. (2021). Myelination of regenerating optic nerve axons occurs in conjunction with an increase of the oligodendrocyte precursor cell population in the adult mice. Brain Research Bulletin, 166, 150–160. 10.1016/j.brainresbull.2020.11.012 33232742

[cne25440-bib-0033] Merchán, P. , Bribián, A. , Sánchez‐Camacho, C. , Lezameta, M. , Bovolenta, P. , & de Castro, F. (2007). Sonic hedgehog promotes the migration and proliferation of optic nerve oligodendrocyte precursors. Molecular and Cellular Neuroscience, 36(3), 355–368. 10.1016/j.mcn.2007.07.012 17826177

[cne25440-bib-0034] Mercurio, S. , Serra, L. , Motta, A. , Gesuita, L. , Sanchez‐Arrones, L. , Inverardi, F. , Foglio, B. , Barone, C. , Kaimakis, P. , Martynoga, B. , Ottolenghi, S. , Studer, M. , Guillemot, F. , Frassoni, C. , Bovolenta, P. , & Nicolis, S. K. (2019). Sox2 acts in thalamicneurons to control the development of retina‐thalamus‐cortex connectivity. iScience, 15, 257–273. 10.1016/j.isci.2019.04.030 31082736 PMC6517317

[cne25440-bib-0035] Modzelewska, K. , Boer, E. F. , Mosbruger, T. L. , Picard, D. , Anderson, D. , Miles, R. R. , Kroll, M. , Oslund, W. , Pysher, T. J. , Schiffman, J. D. , Jensen, R. , Jette, C. A. , Huang, A. , & Stewart, R. A. (2016). MEK inhibitors reverse growth of embryonal brain tumors derived from oligoneural precursor cells. Cell Reports, 17(5), 1255–1264. 10.1016/j.celrep.2016.09.081 27783941

[cne25440-bib-0036] Morcos, Y. , & Chan‐Ling, T. (1997). Identification of oligodendrocyte precursors in the myelinated streak of the adult rabbit retina in vivo. Glia, 21(2), 163–182. 9336232

[cne25440-bib-0037] Münzel, E. J. , Becker, C. G. , Becker, T. , & Williams, A. (2014). Zebrafish regenerate full thickness optic nerve myelin after demyelination, but this fails with increasing age. Acta Neuropathologica Communications, 2, 77. 10.1186/s40478-014-0077-y 25022486 PMC4164766

[cne25440-bib-0038] Münzel, E. J. , Schaefer, K. , Obirei, B. , Kremmer, E. , Burton, E. A. , Kuscha, V. , Becker, C. G. , Brösamle, C. , Williams, A. , & Becker, T. (2012). Claudin k is specifically expressed in cells that form myelin during development of the nervous system and regeneration of the optic nerve in adult zebrafish. Glia, 60(2), 253–270. 10.1002/glia.21260 22020875

[cne25440-bib-0039] Nakamura, K. , Harada, C. , Namekata, K. , & Harada, T. (2006). Expression of olig2 in retinal progenitor cells. Neuroreport, 17(4), 345–349. 10.1097/01.wnr.0000203352.44998.6b 16514356

[cne25440-bib-0040] Nakazawa, T. , Tachi, S. , Aikawa, E. , & Ihnuma, M. (1993). Formation of the myelinated nerve fiber layer in the chicken retina. Glia, 8(2), 114–121. 10.1002/glia.440080207 7691736

[cne25440-bib-0041] Nawaz, S. , Schweitzer, J. , Jahn, O. , & Werner, H. B. (2013). Molecular evolution of myelin basic protein, an abundant structural myelin component. Glia, 61(8), 1364–1377. 10.1002/glia.22520 24040667

[cne25440-bib-0042] Ono, K. , Yasui, Y. , Rutishauser, U. , & Miller, R. H. (1997). Focal ventricular origin and migration of oligodendrocyte precursors into the chick optic nerve. Neuron, 19(2), 283–292. 10.1016/s0896-6273(00)80939-3 9292719

[cne25440-bib-0043] Ono, K. , Yoshii, K. , Tominaga, H. , Gotoh, H. , Nomura, T. , Takebayashi, H. , & Ikenaka, K. (2017). Oligodendrocyte precursor cells in the mouse optic nerve originate in the preoptic area. Brain Structure and Function, 222(5), 2441–2448. 10.1007/s00429-017-1394-2 28293728

[cne25440-bib-0044] Park, H. C. , Mehta, A. , Richardson, J. S. , & Appel, B. (2002). olig2 is required for zebrafish primary motor neuron and oligodendrocyte development. Developmental Biology, 248(2), 356–368. 10.1006/dbio.2002.0738 12167410

[cne25440-bib-0045] Parrilla, M. , León‐Lobera, F. , Lillo, C. , Arévalo, R. , Aijón, J. , Lara, J. M. , & Velasco, A. (2016). Sox10 expression in goldfish retina and optic nerve head in controls and after the application of two different lesion paradigms. PLoS ONE, 11(5), e0154703. 10.1371/journal.pone.0154703 27149509 PMC4858161

[cne25440-bib-0046] Parrilla, M. , Lillo, C. , Herrero‐Turrión, M. J. , Arévalo, R. , Aijón, J. , Lara, J. M. , & Velasco, A. (2012). Characterization of pax2 expression in the goldfish optic nerve head during retina regeneration. PLoS ONE, 7(2), e32348. 10.1371/journal.pone.0032348 22384226 PMC3288081

[cne25440-bib-0047] Parrilla, M. , Lillo, C. , Herrero‐Turrión, M. J. , Arévalo, R. , Aijón, J. , Lara, J. M. , & Velasco, A. (2013). Pax2+ astrocytes in the fish optic nerve head after optic nerve crush. Brain Research, 1492, 18–32. 10.1016/j.brainres.2012.11.014 23165116

[cne25440-bib-0048] Perry, V. H. , & Lund, R. D. (1990). Evidence that the lamina cribrosa prevents intraretinal myelination of retinal ganglion cell axons. Journal of Neurocytology, 19(2), 265–272. 10.1007/BF01217304 2358833

[cne25440-bib-0049] Pinzon‐Olejua, A. , Welte, C. , Chekuru, A. , Bosak, V. , Brand, M. , Hans, S. , & Stuermer, C. A. (2017). Cre‐inducible site‐specific recombination in zebrafish oligodendrocytes. Developmental Dynamics, 246(1), 41–49. 10.1002/dvdy.24458 27666728

[cne25440-bib-0050] Preston, M. A. , & Macklin, W. B. (2015). Zebrafish as a model to investigate CNS myelination. Glia, 63(2), 177–193. 10.1002/glia.22755 25263121 PMC4539269

[cne25440-bib-0051] Ravanelli, A. M. , Kearns, C. A. , Powers, R. K. , Wang, Y. , Hines, J. H. , Donaldson, M. J. , & Appel, B. (2018). Sequential specification of oligodendrocyte lineage cells by distinct levels of hedgehog and notch signaling. Developmental Biology, 444(2), 93–106. 10.1016/j.ydbio.2018.10.004 30347186 PMC6263812

[cne25440-bib-0052] Reichenbach, A. , Schippel, K. , Schümann, R. , & Hagen, E. (1988). Ultrastructure of rabbit retinal nerve fibre layer–neuro‐glial relationships, myelination, and nerve fibre spectrum. Journal Fur Hirnforschung, 29(5), 481–491. 3216098

[cne25440-bib-0053] Sagner, A. , Gaber, Z. B. , Delile, J. , Kong, J. H. , Rousso, D. L. , Pearson, C. A. , Weicksel, S. E. , Melchionda, M. , Gharavy, S. N. M. , Briscoe, J. , & Novitch, B. G. (2018). Olig2 and Hes regulatory dynamics during motor neuron differentiation revealed by single cell transcriptomics. Plos Biology, 16(2), e2003127. 10.1371/journal.pbio.2003127 29389974 PMC5811045

[cne25440-bib-0054] Santos, E. , Yanes, C. M. , Monzón‐Mayor, M. , & Romero‐Alemán, M. D. M. (2006). Peculiar and typical oligodendrocytes are involved in an uneven myelination pattern during the ontogeny of the lizard visual pathway. Journal of Neurobiology, 66(10), 1115–1124. 10.1002/neu.20256 16929522

[cne25440-bib-0055] Santos‐Ledo, A. , Arenzana, F. J. , Porteros, A. , Lara, J. , Velasco, A. , Aijón, J. , & Arévalo, R. (2011). Cytoarchitectonic and neurochemical differentiation of the visual system in ethanol‐induced cyclopic zebrafish larvae. Neurotoxicology and Teratology, 33(6), 686–697. 10.1016/j.ntt.2011.06.001 21684331

[cne25440-bib-0056] Santos‐Ledo, A. , Garcia‐Macia, M. , Campbell, P. D. , Gronska, M. , & Marlow, F. L. (2017). Kinesin‐1 promotes chondrocyte maintenance during skeletal morphogenesis. Plos Genetics, 13(7), e1006918. 10.1371/journal.pgen.1006918 28715414 PMC5536392

[cne25440-bib-0057] Shibasaki, K. , Takebayashi, H. , Ikenaka, K. , Feng, L. , & Gan, L. (2007). Expression of the basic helix‐loop‐factor olig2 in the developing retina: Olig2 as a new marker for retinal progenitors and late‐born cells. Gene Expression Patterns, 7(1–2), 57–65. 10.1016/j.modgep.2006.05.008 16815098

[cne25440-bib-0058] Shin, J. , Park, H. C. , Topczewska, J. M. , Mawdsley, D. J. , & Appel, B. (2003). Neural cell fate analysis in zebrafish using olig2 BAC transgenics. Methods in Cell Science, 25(1–2), 7–14. 10.1023/B:MICS.0000006847.09037.3a 14739582

[cne25440-bib-0059] Takada, N. , & Appel, B. (2010). Identification of genes expressed by zebrafish oligodendrocytes using a differential microarray screen. Developmental Dynamics, 239(7), 2041–2047. 10.1002/dvdy.22338 20549738

[cne25440-bib-0060] Takada, N. , Kucenas, S. , & Appel, B. (2010). Sox10 is necessary for oligodendrocyte survival following axon wrapping. Glia, 58(8), 996–1006. 10.1002/glia.20981 20229602 PMC3639140

[cne25440-bib-0061] Thummel, R. , Kassen, S. C. , Enright, J. M. , Nelson, C. M. , Montgomery, J. E. , & Hyde, D. R. (2008). Characterization of müller glia and neuronal progenitors during adult zebrafish retinal regeneration. Experimental Eye Research, 87(5), 433–444. 10.1016/j.exer.2008.07.009 18718467 PMC2586672

[cne25440-bib-0062] Tian, C. , Zou, S. , & Hu, B. (2016). Extraocularsource of oligodendrocytes contribute to retinal myelination and optokinetic responses in zebrafish. Investigative Ophthalmology & Visual Science, 57(4), 2129–2138. 10.1167/iovs.15-17675 27100159

[cne25440-bib-0063] Tiwari, S. , Dharmarajan, S. , Shivanna, M. , Otteson, D. C. , & Belecky‐Adams, T. L. (2014). Histone deacetylase expression patterns in developing murine optic nerve. BMC Developmental Biology, 14, 30. 10.1186/1471-213X-14-30 25011550 PMC4099093

[cne25440-bib-0064] Wegner, M. , & Stolt, C. C. (2005). From stem cells to neurons and glia: Asoxist's view of neural development. Trends in Neuroscience, 28(11), 583–588. 10.1016/j.tins.2005.08.008 16139372

[cne25440-bib-0065] Xiao, Y. , Petrucco, L. , Hoodless, L. J. , Portugues, R. , & Czopka, T. (2022). Oligodendrocyte precursor cells sculpt the visual system by regulating axonal remodeling. Nature Neuroscience, 25(3), 280–284. 10.1038/s41593-022-01023-7 35241802 PMC8904260

[cne25440-bib-0066] Zou, S. , & Hu, B. (2021). In vivo imaging reveals mature oligodendrocyte division in adult zebrafish. Cell Regeneration, 10(1), 16. 10.1186/s13619-021-00079-3 34075520 PMC8169745

